# Factors associated with non-utilization of child immunization in Pakistan: evidence from the Demographic and Health Survey 2006-07

**DOI:** 10.1186/1471-2458-14-232

**Published:** 2014-03-06

**Authors:** Ayesha Siddiqa Bugvi, Rahla Rahat, Rubeena Zakar, Muhammad Zakria Zakar, Florian Fischer, Muazzam Nasrullah, Riffat Manawar

**Affiliations:** 1 Institute of Social and Cultural Studies, University of the Punjab, Lahore, Pakistan; 2 Department of Public Health Medicine, School of Public Health, Bielefeld University, Bielefeld, Germany; 3 Rollins School of Public Health, Emory University, Atlanta, Georgia, USA; 4 Injury Control Research Center, West Virginia University, Morgantown, West Virginia, USA

**Keywords:** Childhood diseases, Immunization, Pakistan, Demographic and Health Survey

## Abstract

**Background:**

The proportion of incompletely immunized children in Pakistan varies from 37-58%, and this has recently resulted in outbreaks of measles and polio. The aim of this paper is to determine the factors associated with incomplete immunization among children aged 12-23 months in Pakistan.

**Methods:**

Secondary analysis was conducted on nationally representative cross-sectional survey data from the Pakistan Demographic and Health Survey, 2006-07. The analysis was limited to ever-married mothers who had delivered their last child during the 23 months immediately preceding the survey (n = 2,435). ‘Complete immunization’ was defined as the child having received twelve doses of five vaccines, and ‘incomplete immunization’ was defined if he/she had missed at least one of these twelve doses. The association between child immunization status and determinants of non-utilization of vaccines was assessed by calculating unadjusted and adjusted odds ratios (AOR) with 95% confidence intervals using a multivariable binary logistic regression.

**Results:**

The findings of this research showed that nearly 66% of children were incompletely immunized against seven preventable childhood diseases. The likelihood of incomplete immunization was significantly associated with the father’s occupation as a manual worker (AOR = 1.47; 95% CI: 1.10-1.97), lack of access to information (AOR = 1.35; 95% CI: 1.09-1.66), non-use of antenatal care (AOR = 1.33; 95% CI: 1.07-1.66), children born in Baluchistan region (AOR = 1.74; 95% CI: 1.12-2.70) and delivery at home (AOR = 1.39; 95% CI: 1.14-1.69).

**Conclusions:**

Despite governmental efforts to increase rates of immunization against childhood diseases, the proportion of incompletely immunized children in Pakistan is still high. Targeted interventions are needed to increase the immunization rates in Pakistan. These interventions need to concentrate on people with low socioeconomic and educational status in order to improve their knowledge of this topic.

## Background

Vaccination is one of the major contributors to public health. It has eliminated some of the most dreaded childhood diseases, such as polio, from all over the world except in a few countries [1]. Childhood immunization is considered to be one of the most important health indicators of a healthy childhood. It assures protection from major childhood diseases, is estimated to prevent millions of deaths and cases of disability worldwide, and is therefore considered beneficial by the scientific community [2].

In 1974, the World Health Organization (WHO) launched its Expanded Program on Immunization (EPI) with the aim of controlling six childhood diseases: tuberculosis, diphtheria, pertussis (whooping cough), tetanus, polio and measles. Consistent with the WHO’s recommendations, Pakistan launched its EPI in 1978 under the guidance of the WHO [3]. Currently, the national EPI aims to immunize all children between the ages of 0 and 23 months against eight vaccine-preventable diseases which include, in addition to the above-mentioned diseases, hepatitis B (vaccine introduced in 2002) and haemophilus influenza type b (Hib) (vaccine introduced in 2008) [3] (Table 1).

**Table 1:** Routine immunization schedule in Pakistan

**Age of child**	**Vaccination**
At birth	BCG and Polio 0
6 weeks	DPT1 + HBV1 + Polio1 + Hib1
10 weeks	DPT2 + HBV2 + Polio2 + Hib2
14 weeks	DPT3 + HBV3 + Polio3 + Hib3
9 months	Measles
Second year of life	Measles

Source: Immunization in Pakistan [3].

In Pakistan, the Health Department provides the immunization schedule for the national EPI in accordance with the WHO guidelines. The EPI is funded by the United Nations International Children’s Fund (UNICEF). It is provided free of charge at all state-run health facilities, which are present in every district across Pakistan. Despite having a national immunization program, and a quarterly anti-polio campaign, Pakistan is still one of the few countries that is not polio free [4]. The WHO confirmed that in Pakistan there were 198 polio cases in 2011 [5], 58 in 2012 and 83 cases were reported in 2013 [6]. Pakistan has also seen a rise in measles cases in the past couple of years. In 2011, 64 children died, and in 2012 the number increased to 306, with Sindh as the most affected province [7]. It is reported that there were 192 deaths from measles in the Punjab province of Pakistan in 2013 [8].

Although the trends in immunization coverage show improvement for individual doses, especially polio, the percentage of fully immunized children aged 12-23 months who have received specific vaccines was only 47.3% [3]. Many studies have examined the factors that affect immunization rates amongst children. Most of them looked at determinants of utilization of child immunization. Among them, some have looked at individual factors, such as the education, knowledge, attitude and practice of mothers regarding immunization [9-12], while a few focused on structural issues, such as barriers to immunization [13,14].

The benefits of getting a child immunized are hardly questionable, yet studies have shown that, in many developing countries, a sizeable number of parents, usually belonging to socioeconomically disadvantaged populations, resist child immunization [9,13]. The primary reason for parents not getting their children immunized is the perception that their children will not be infected with certain diseases such as polio, whooping cough and measles. Further, the parents are apprehensive about the side effects of immunization [15]. Due to structural, cultural and economic factors, Pakistan has lower immunization coverage than other countries in the region [4]. To our knowledge, very few studies [16,17] have looked at the determinants of non-utilization of child immunization in Pakistan, although some have looked at determinants of utilization of specific vaccines [14,18,19]. Our study aims to describe the individual factors associated with incomplete immunization among last-born children aged 12-23 months in Pakistan.

## Methods

Secondary data analysis was conducted on data from the Pakistan Demographic and Health Survey (PDHS) 2006-07. The PDHS is a publicly available dataset produced by ORC Macro for the Measure DHS (Demographic and Health Surveys) Project. It is funded by the US Agency for International Development (USAID). The PDHS contains a wide range of health-related information and is considered to be free of systematic bias [20].

The PDHS is the largest household survey conducted in Pakistan. It is comprised of a nationally representative sample of over 95,000 households collected using a stratified two-stage cluster sampling procedure. However, for security and political reasons, some areas, such as Federally Administered Tribal Areas (FATA), Federally Administered National Areas (FANA) and Azad Jammu and Kashmir (AJK), were not part of the survey. Amongst several modules on fertility, nutrition, reproductive health and malaria in the PDHS, one is on child immunization [21]. The detailed methodology of the survey design, data collection and management has been described elsewhere [21].

Our data was limited to mothers with a last-born child (youngest child) between the ages of 12 to 23 months, resulting in a sample size of 2,435. The reason for the selection of this age group was that, until 2006, the course of basic vaccinations (i.e., 12 doses for seven vaccine-preventable diseases) for children was completed by the age of 9 months. Some previous studies have also used the same age-group for studying the utilization of immunization [10,11,14]. We computed the dependent variable, “immunization status” by using twelve doses of 5 vaccines, i.e. polio (4 doses), BCG (1 dose), DPT (3 doses), HBV (3 doses) and measles (1 dose). The data on Haemophilus influenza type b (Hib) vaccination were not available in the PDHS 2006-07, as it was first introduced in 2008 in the national EPI. Similarly, no information was available on the Measles 2 vaccination, as it was added to the routine immunization schedule in Pakistan in 2012.

We selected twelve variables, which showed “Received: BCG, polio (0,1,2,3), DPT (1,2,3), HBV (1,2,3) and measles”. These variables had five response categories: No, vaccination date on card, reported by mother, vaccination marked on card and DK (don’t know). We recoded each variable in a similar way. No and DK responses were recoded as “0” and considered as “not received the vaccine”, while the other responses “vaccination date on card, reported by mother, vaccination marked on card” were recoded as “1” and considered as “received the vaccine”. Later, we added all twelve vaccine variables and labeled them “Immunization status”. The immunization status was recoded as “0” if the child had received all twelve doses of the above-mentioned vaccinations and categorized as “complete immunization”, and “1” if the child had missed one or more vaccinations, and categorized as “incomplete immunization”.

We defined “complete immunization” in accordance with the WHO, which considers a child completely immunized if “he or she has received a BCG vaccination against tuberculosis; three doses of DPT vaccine to prevent diphtheria, pertussis, and tetanus (DPT); at least three doses of polio vaccine; and one dose of measles vaccine.” Previously published studies have defined “complete immunization” in a similar way [22,23].

Based on a literature review [9-15] and available data within the PDHS 2006-07, 14 independent variables were identified. These were: mother’s age, father’s age, mother’s education, father’s education, father’s occupation, wealth index (measured on the basis of household assets and ownership of a number of consumer items and divided into quintiles from one [poorest] to five [richest]), sex of child, birth order of child, place of residence, region, access to information, seeking formal advice/treatment, use of antenatal care and place of delivery. The independent variables, such as sex of child, place of residence (urban/rural) and region (Sindh, Baluchistan, North Western Frontiers Province vs. Punjab [because Punjab is the most developed region having good social indicators]) were used as such. Some independent variables were also recoded, such as mother’s and father’s age (15-24, 25-34 and ≥ 35), mother’s and father’s education (no education, up to primary, up to secondary and higher), father’s occupation (not working, manual worker, clerical/sales/service and management/professional),wealth index (poor, middle and rich) and birth order of the child (1-3, 4-6 and ≥ 7).

The remaining independent variables were computed and recoded, and limited to categories between two and four because of the small number of cases in some categories. The variable: “seeking formal advice/treatment” was computed using three variables from the PDHS, a) Seek advice/treatment from doctor, b) Seek advice/treatment from Midwife/LHV/nurse and c) Seek advice/treatment from Lady Health Worker (LHW). After computation, the variable was recoded into “0” if the mother has not taken advice/treatment from any of these sources and categorized as “no”, and “1” if the mother has taken advice from one or more of these sources and categorized as “yes”. Similarly, the variable “access to information” was computed and recoded using three variables: a) Access to radio, b) Access to television and c) Access to computer. The data was recoded into “0” if the mother has no access to any of these sources and categorized as “no”, and “1” if the mother had access to one or more of the above-mentioned sources and categorized as “yes”.

### Statistical analysis

All the data were weighted and analyzed using SPSS version 17 to account for selection probability, non-response, and sampling differences between regions to produce national estimates of the population. Descriptive statistics for both groups (incomplete immunization and complete immunization) were presented as frequency distributions and percentages. Simple binary logistic regression analysis was carried out to examine the relationship between “incomplete immunization” and the independent variables. In the multiple logistic model, three variables (mother’s age, mother’s education, wealth quintile) were adjusted and fixed. We entered all the independent variables that were significant at the 0.05 level one by one into the model. We also assessed the multicollinearity between the variables and highly correlated variables were eliminated from the logistical model. Multicollinearity was assessed between mother’s and father’s age through Pearson correlation and it was significant at the 0.01 level, so the father’s age variable was eliminated from the model.

### Ethical considerations

This study is based on secondary analysis of publicly available data; hence no ethical approval was required from our institutions. Permission to use the PDHS-2006-07 data was obtained from Measure DHS.

## Results

### Individual characteristics and immunization status of children

The mean age of mothers and fathers was 29.42 (SD ± 6.49) years and 36.99 (SD ± 10.0) years respectively. Two thirds of the selected children (66%) were incompletely immunized. The proportion of incompletely immunized was slightly higher among female children (68.6%) than among male children (64.0%) (Table 2). Younger (15-24 years) and older (≥ 35 years) mothers had the highest percentages of incompletely immunized children; however, amongst fathers, only the younger group (15-24 years) had the highest percentage of incompletely immunized children (80.8%). Parents who were illiterate had the highest percentage of incompletely immunized children. Children with higher birth order (> 7) were more likely to be incompletely immunized (74.4%). As far as regional differences were concerned, more than three quarters of children in the province of Baluchistan were incompletely immunized (79.9%). Lastly, of those children who were delivered at home, 73.6% were incompletely immunized, and of those born to mothers who had not used antenatal services, 77.2% were incompletely immunized.

**Table 2:** **Individual characteristics and immunization status of children aged 12**-**23 months**

**Variables**	**Participants (N** **=** **2435)**		
	**Immunization status**		
	**Completely immunized**	**Incompletely immunized**	
	**n** **=** **825**	**n** **=** **1610**	
	**N (Weighted%)**	**N (Weighted%)**	**Total**
**Mother’s age (years)**			
15-24	191 (32.4)	398 (67.6)	589
25-34	462 (35.4)	843 (64.6)	1306
≥ 35	172 (31.7)	369 (68.3)	540
**Father’s age (years)**			
15-24	34 (19.2)	142 (80.8)	176
25-34	359 (35.4)	655 (64.6)	1014
≥ 35	423 (34.9)	790 (65.1)	1213
**Mother’s education**			
No education	397 (25.8)	1138 (74.2)	1535
Up to primary	126 (36.8)	218 (63.2)	345
Up to secondary	190 (50.5)	186 (49.5)	376
Higher	112 (62.1)	68 (37.9)	180
**Father’s education**			
No education	184 (22.1)	648 (77.9)	832
Up to primary	104 (27.3)	277 (72.7)	381
Up to secondary	333 (42.3)	454 (57.7)	787
Higher	204 (46.8)	231 (53.2)	435
**Father’s occupation**			
Not working	27 (31.8)	57 (68.2)	84
Manual worker	406 (28.9)	997 (71.1)	1403
Clerical/sales/service	266 (38.5)	425 (61.5)	691
Management/professional	125 (48.9)	131 (51.1)	256
**Place of residence**			
Rural	436 (28.8)	1076 (71.2)	1512
Urban	389 (42.2)	534 (57.8)	923
**Sex of child**			
Female	348 (31.4)	761 (68.6)	1109
Male	477 (36.0)	849 (64.0)	1326
**Birth order**			
≥ 7	96 (25.6)	280 (74.4)	376
4-6	261 (32.6)	539 (67.4)	800
1-3	468 (37.2)	791 (62.8)	1260
**Wealth index**			
Poor	197 (19.3)	824 (80.7)	1021
Middle	368 (39.7)	559 (60.3)	927
Rich	260 (53.4)	227 (46.6)	487
**Seek formal advice/treatment**			
No	244 (30.2)	572 (69.8)	816
Yes	323 (36.7)	546 (63.3)	869
**Access to information**			
No	197 (23.2)	650 (76.8)	847
Yes	625 (39.5)	955 (60.5)	1580
**Region**			
Sindh	245 (28.1)	627 (71.9)	872
North Western Frontier Province^†^	158 (43.0)	210 (57.0)	368
Baluchistan	29 (20.1)	117 (79.9)	146
Punjab	392 (37.4)	657 (62.6)	1049
**Place of delivery**			
Home	385 (26.4)	1076 (73.6)	1461
Hospital	440 (45.2)	533 (54.8)	973
**Use of antenatal care**			
No	175(22.8)	529 (77.2)	767
Yes	646 (39.2)	1001 (60.8)	1646

Absolute number of participants does not perfectly correspond to percentages because the percentages are weighted.

^†^Now called Khyber Pakhtoon Khawa.

### **Determinants of non**-**immunization**

#### ***Simple binary logistic regression analysis***

The data showed that children of poor parents (OR = 4.78; 95% CI: 3.78-6.06) were more likely to be incompletely immunized (Table 3). The chances of incomplete immunization were high for children born to illiterate fathers (OR = 3.09; 95% CI: 2.41-3.98), younger fathers (OR = 2.25; 95% CI: 1.52-3.33) and fathers employed as manual workers (OR = 2.36; 95% CI: 1.80-3.09). Similarly, the odds of incomplete immunization were high for children of mothers who had no formal education (OR = 4.71; 95% CI: 3.41-6.50), had no access to information (OR = 2.16; 95% CI: 1.79-2.61), and had not used antenatal care (OR = 2.18; 95% CI: 1.79-2.65). Additionally, children were more likely to be incompletely immunized when born in Baluchistan region (OR = 2.37; 95% CI: 1.55-3.62), had birth order 4-6 (middle birth order) (OR = 1.72; 95% CI: 1.33-2.22) or were delivered at home (OR = 2.30; 95% CI: 1.94-2.74).

**Table 3:** **Simple Binary logistic regression for the predictors associated with incomplete immunization among children aged 12**-**23 months**

**Variables**	**Incomplete immunization**	**P value**
	**OR (95% CI)**	
**Mother’s age (years)**		
15-24	0.97 (0.75-1.24)	0.801
25-34	0.85 (0.68-1.05)	0.134
≥ 35	1	
**Father’s age (years)**		
15-24	2.25 (1.52-3.33)	< 0.001
25-34	0.98 (0.82-1.17)	0.812
≥ 35	1	
**Mother’s education**		
No education	4.71 (3.41-6.50)	< 0.001
Up to primary	2.82 (1.94-4.09)	< 0.001
Up to secondary	1.61 (1.12-2.32)	0.009
Higher	1	
**Father’s education**		
No education	3.09 (2.41-3.98)	< 0.001
Up to primary	2.35 (1.75-3.15)	< 0.001
Up to secondary	1.20 (095-1.52)	0.133
Higher	1	
**Father’s occupation**		
Not working	2.06 (1.22-3.46)	0.007
Manual worker	2.36 (1.80-3.09)	< 0.001
Clerical/sales/service	1.53 (1.15-2.04)	0.004
Management/professional	1	
**Place of residence**		
Rural	1.80 (1.52-2.14)	< 0.001
Urban	1	
**Sex of child**		
Female	1.23 (1.04-1.46)	0.017
Male	1	
**Birth order**		
≥ 7	1.22 (1.02-1.48)	0.035
4-6	1.72 (1.33-2.22)	< 0.001
1-3	1	
**Wealth index**		
Poor	4.78 (3.78-6.06)	< 0.001
Middle	1.74 (1.40-2.18)	< 0.001
Rich	1	
**Seek formal advice/treatment**		
No	1.39 (1.13-1.70)	0.002
Yes	1	
**Access to information**		
No	2.16 (1.79-2.61)	< 0.001
Yes	1	
**Region**		
Sindh	1.52 (1.26-1.85)	< 0.001
North Western Frontier Province^†^	0.79 (0.62-1.01)	0.055
Baluchistan	2.37 (1.55-3.62)	< 0.001
Punjab	1	
**Place of delivery**		
Home	2.30 (1.94-2.74)	< 0.001
Hospital	1	
**Use of antenatal care**		
No	2.18 (1.79-2.65)	< 0.001
Yes	1	

*Abbreviations*: 1, reference category; OR, odds ratio; CI, confidence interval;

^†^Now called Khyber Pakhtoon Khawa.

#### ***Multivariable logistic regression analysis***

After adjusting for mother’s age, education and wealth quintile, the mothers who lacked access to information (AOR = 1.35; 95% CI: 1.09-1.66), had not used antenatal care (AOR = 1.33; 95% CI: 1.07-1.66) or delivered at home (AOR = 1.39; 95% CI: 1.14-1.69) were more likely to have children with incomplete immunization (Table 4). Children born in Baluchistan region were 1.7 times more likely to be incompletely immunized (AOR = 1.74; 95% CI: 1.12-2.70) than children born in Punjab. Similarly, the odds of incomplete immunization were high for children born to manual workers (AOR = 1.47; 95% CI: 1.10-1.97) compared to those whose fathers were professionals or had managerial jobs.

**Table 4:** **Adjusted odds ratio for multivariable logistic regression of factors independently associated with incomplete immunization among children aged 12**-**23 months**

**Variables**	**Incomplete immunization**	**P value**
	**AOR (95% CI)**	
**Father’s occupation**		
Not working	1.27 (0.74-2.19)	0.391
Manual worker	1.47 (1.10-1.97)	0.009
Clerical/sales/service	1.28 (0.94-1.74)	0.112
Management/professional	1	
**Place of residence**		
Rural	0.89 (0.72-1.09)	0.262
Urban	1	
**Birth order**		
≥ 7	1.28 (0.91-1.82)	0.161
4-6	1.07 (0.85-1.35)	0.554
1-3	1	
**Seek formal advice/treatment**		
No	1.07 (0.86-1.33)	0.553
Yes	1	
**Access to information**		
No	1.35 (1.09-1.66)	0.006
Yes	1	
**Region**		
Sindh	1.35 (1.09-1.65)	0.004
North Western Frontier Province†	0.59 (0.46-0.76)	< 0.001
Baluchistan	1.74 (1.12-2.70)	0.014
Punjab	1	
**Use of antenatal care**		
No	1.33 (1.07-1.66)	0.009
Yes	1	
**Place of delivery**		
Home	1.39 (1.14-1.69)	0.001
Hospital	1	

*Abbreviations*: 1, reference category; AOR, adjusted odds ratio; CI, confidence interval.

A Multivariable logistic regression analysis was carried out to obtain the AOR after controlling for mother’s age (continuous variable), mother’s education and wealth quintile.

^†^Now called Khyber Pakhtoon Khawa.

## Discussion

Incomplete immunization is one of the major child health issues in Pakistan. Our study found that the children of manual workers were at higher risk of incomplete immunization than the children of relatively better off professionals. As in an earlier study that showed an association between type of maternal occupation (e.g. unskilled workers) and incomplete child immunization [24], it is likely that poorly paid and poorly educated manual workers may not find the time or resources to travel to their nearby health facility for immunization. Additionally, because of parents’ poor “health literacy” [25], they may not be able to properly understand the preventive benefits of timely and complete immunization.

Information could play a pivotal role in determining the health behavior of an individual. We found that lack of access to information among mothers increases the likelihood of incomplete immunization for their children. In Pakistan generally, public awareness about immunization, especially among mothers from poor socioeconomic strata, is very low. Therefore, the government of Pakistan dedicates a few days every year to the National Immunization Campaign to increase the level of awareness and motivation about timely completion of the immunization process for children.

Similarly, the level of social development of a region could influence the extent to which parents can avail themselves of preventive health services. Our study showed that region of residence is associated with incomplete immunization. For instance, children in relatively less developed provinces, such as Sindh and Baluchistan, were more likely to have incomplete immunization than those in Punjab, which is more socially and economically developed. Even in North Western Frontier province, now called Khyber Pakhtoon Khawa, complete immunization is much better than other regions because many international organizations such as UNICEF, USAID, the World Bank, UN Women and national organizations such as the Aurat Foundation, PAIMAN and others are working to improve maternal and child health.

Immunization may not be an isolated phenomenon; its timely dispensation is linked with parents’ previous interactions with the health-care system [13,24]. For example, if a pregnant mother has antenatal visits, she may get information about immunization and become acquainted with health-care staff. Such acquaintance may be very helpful in getting the child immunized after delivery. Our data also showed that mothers who had infrequent or no antenatal visits had a high probability of incomplete immunization for their children. Similarly, mothers who delivered at home also had a lower chance of complete immunization. The reason could be that the mothers who had home deliveries may have had weaker or no acquaintance with health-care staff and hence were less aware of the importance of the timely completion of vaccination [10].

In patriarchal societies, including Pakistan, a male baby is more valued than a female baby, because males are considered to have economic and social utility in families [26]. However, our study did not find any difference between male and female child immunization status. The reason could be that parents may not be aware of the preventive benefits of immunization. Hence, they may not realize the importance of the completion of immunization for their children regardless of their sex. Although a few studies have found that gender can be a relevant factor in the completion of child immunization [22,27], our analysis did not provide this evidence and it was consistent with other studies which also found no association of child immunization with either sex [24,28].

### Limitations

The above findings ought to be considered in light of certain limitations: Firstly, the analysis was restricted to the last child born during the 23 months immediately preceding the survey; hence, it cannot be generalized to all children under five years of age. Secondly, the analysis conducted cannot establish causal relationships between immunization status and any of the independent variables because of the cross-sectional nature of the survey. Lastly, information on child immunization in the survey is based on either immunization cards or the self-reports of women and the information received through self-reports is subject to recall and social desirability biases. Due to this fact, the completeness of vaccination might be overestimated, because self-reports by the mother were handled in the same way as information from vaccination cards. Additionally, a binary categorization (vaccination received vs. not received) was used, which may lead to a loss of information.

## Conclusions

The rate of incomplete immunization of children in Pakistan is alarming. Father’s occupation as an unskilled worker, residence in less developed regions such as Baluchistan, lack of access to health information, non-use of antenatal care and home deliveries were found to be independently associated with incomplete immunization of children in Pakistan. Despite limitations, this study shows that EPI services are not reaching the target population and efforts should be made to provide full coverage of vaccination to all children in Pakistan. Therefore, modified interventions are needed to increase the rates of fully immunized children in Pakistan. Additionally, a longitudinal study is needed to explore the factors associated with incomplete immunization levels for each vaccination.

## Competing interests

The authors declare that they have no competing interests.

## Authors’ contributions

ASB and RR drafted the initial manuscript. RZ, MZZ and MN supervised and reviewed manuscript writing. ASB and RR contributed in data analysis while RZ supervised data analysis. FF and RM contributed to reviewing final manuscript. All authors have read and approved the final manuscript.

## Pre-publication history

The pre-publication history for this paper can be accessed here:

http://www.biomedcentral.com/1471-2458/14/232/prepub
